# In vitro recellularization of decellularized bovine carotid arteries using human endothelial colony forming cells

**DOI:** 10.1186/s13036-021-00266-5

**Published:** 2021-04-21

**Authors:** Nicolai Seiffert, Peter Tang, Eriselda Keshi, Anja Reutzel-Selke, Simon Moosburner, Hannah Everwien, Dag Wulsten, Hendrik Napierala, Johann Pratschke, Igor M. Sauer, Karl H. Hillebrandt, Benjamin Struecker

**Affiliations:** 1 Charité – Universitätsmedizin Berlin, corporate member of Freie Universität Berlin and Humboldt- Universität zu Berlin, Department of Surgery, Campus Charité Mitte | Campus Virchow-Klinikum, Augustenburger Platz 1, 13353 Berlin, Germany; 2 Department for Trauma and Orthopedic Surgery, Vivantes-Hospital Spandau, Berlin, Germany; 3 Berlin Institute of Health at Charité – Universitätsmedizin Berlin, Charitéplatz 1, 10117 Berlin, Germany; 4 Charité – Universitätsmedizin Berlin, corporate member of Freie Universität Berlin and Humboldt- Universität zu Berlin, Julius Wolff Institute, Augustenburger Platz 1, 13353 Berlin, Germany; 5 Berlin Institute of Health at Charité – Universitätsmedizin Berlin, BIH Academy, Clinician Scientist Program, Charitéplatz 1, 10117 Berlin, Germany; 6 Department of General, Visceral and Transplant Surgery, University Hospital Münster, Münster, Germany

**Keywords:** Endothelial Colony forming cells (ECFC), Mesenchymal stromal cells (MSC), Tissue engineering, Bovine carotid artery, Decellularization, Recellularization, Target group specific cells / sick cells, Impaired cell function

## Abstract

**Background:**

Many patients suffering from peripheral arterial disease (PAD) are dependent on bypass surgery. However, in some patients no suitable replacements (i.e. autologous or prosthetic bypass grafts) are available. Advances have been made to develop autologous tissue engineered vascular grafts (TEVG) using endothelial colony forming cells (ECFC) obtained by peripheral blood draw in large animal trials. Clinical translation of this technique, however, still requires additional data for usability of isolated ECFC from high cardiovascular risk patients.

Bovine carotid arteries (BCA) were decellularized using a combined SDS (sodium dodecyl sulfate) -free mechanical-osmotic-enzymatic-detergent approach to show the feasibility of xenogenous vessel decellularization. Decellularized BCA chips were seeded with human ECFC, isolated from a high cardiovascular risk patient group, suffering from diabetes, hypertension and/or chronic renal failure. ECFC were cultured alone or in coculture with rat or human mesenchymal stromal cells (rMSC/hMSC). Decellularized BCA chips were evaluated for biochemical, histological and mechanical properties. Successful isolation of ECFC and recellularization capabilities were analyzed by histology.

**Results:**

Decellularized BCA showed retained extracellular matrix (ECM) composition and mechanical properties upon cell removal. Isolation of ECFC from the intended target group was successfully performed (80% isolation efficiency). Isolated cells showed a typical ECFC-phenotype. Upon recellularization, co-seeding of patient-isolated ECFC with rMSC/hMSC and further incubation was successful for 14 (*n* = 9) and 23 (*n* = 5) days. Reendothelialization (rMSC) and partial reendothelialization (hMSC) was achieved. Seeded cells were CD31 and vWF positive, however, human cells were detectable for up to 14 days in xenogenic cell-culture only. Seeding of ECFC without rMSC was not successful.

**Conclusion:**

Using our refined decellularization process we generated easily obtainable TEVG with retained ECM- and mechanical quality, serving as a platform to develop small-diameter (< 6 mm) TEVG. ECFC isolation from the cardiovascular risk target group is possible and sufficient. Survival of diabetic ECFC appears to be highly dependent on perivascular support by rMSC/hMSC under static conditions. ECFC survival was limited to 14 days post seeding.

**Supplementary Information:**

The online version contains supplementary material available at 10.1186/s13036-021-00266-5.

## Background

Cardiovascular diseases continue to be the leading cause of death in the United States [1]. Peripheral artery disease (PAD), especially in its advanced form critical limb ischemia (CLI), remains a major cause of vascular-related morbidity and mortality [2] while being a burden clinically and economically to western world healthcare systems [3, 4]. Bypass surgery is one of the most commonly applied surgical treatment of PAD today [5, 6], with patient-derived grafts remaining the gold standard to bypass constricted areas. Unfortunately, up to 30% of patients suffering from PAD are unable to provide suitable autologous vessel grafts [7, 8]. Synthetic prosthetic grafts may be used as an alternative for large diameter arteries (> 6 mm), however, mechanical mismatch, adverse host response, reduced patency rates and increased susceptibility to infections have impeded clinical applicability, especially for medium or small diameter arteries (inner diameter ≤ 6 mm) [8–12]. To address these problems and create viable alternatives, tissue engineered vascular grafts (TEVG) have been developed using self-assembly and biodegradable scaffold techniques of both synthetic and natural origin [13]. While the results seem promising and are pursued up to clinical trials, no widespread clinical application has been established so far [13, 14].

Promising, in this context, is the technique of decellularization, whereby all cellular material is removed and the extracellular matrix (ECM) is preserved with favorable mechanical and biochemical properties [13–18]. Decellularized constructs are seen as interesting platforms [19] for cellular repopulation and have been tolerated in xenogeneic applications [20]. Resulting acellular vascular scaffolds from bovine origin have been commercialized and are available for off-the-shelf purchase as alternatives to expanded polytetrafluoroethylene (ePTFE) prostheses for both hemodialysis access and treatment of CLI in cases of infected implantation sites [21, 22]. After cell removal, however, functional endothelium is absent and the underlying vascular ECM is exposed to blood which may cause both early and late graft failure by thrombosis and intimal hyperplasia [23]. As a prevention, reendothelialization should be pursued to mask thrombocyte-activating ECM and to control smooth muscle cell (SMC) proliferation and contractility [24].

Although recellularized grafts using endothelial cells (EC) have shown improved patency (89% seeded vs. 29% acellular) [25] and remodeling capacity [20, 26], the isolation of EC is associated with invasive procedures causing donor site complications, providing a low cell yield and may not be feasible for cell isolation due to vessel quality or availability [27, 28]. Endothelial colony forming cells (ECFC) may be isolated by peripheral blood draw and have successfully been expanded to large-scale in vitro experiments [29–31]. Additionally, ECFC seem not only to be able to cover exposed subendothelial ECM but may actually be of a superior thrombo-protective and intimal hyperplasia limiting phenotype when exposed to shear stress [32, 33]. Moreover, to support and stabilize ECFC the importance of co-culture with mesenchymal stromal cells (MSC) has been emphasized [29, 34]. Also, MSC have shown to be of immunomodulatory characteristic and used successfully in xenogenic application [35–37].

ECFC have been in the focus of research for years [38]. In most cases umbilical cord blood-derived ECFC and ECFC isolated from young and healthy donors have been used, although an altered function of adult peripheral ECFC has been reported [39]. Additionally, mounting evidence suggests altered function and proliferation rate of ECFC from aging patients with high cardiovascular risk, including diabetes, hypertension and chronic renal disease [40–42]. Scaffold-reendothelialization using patient-derived ECFC has only been researched to cover synthetic vascular scaffolds so far [43–45]. To our knowledge, the suitability of endothelial colony forming cells sourced by peripheral blood draw from cardiovascular risk patients, the intended clinical target group, for reendothelialization of decellularized vascular scaffolds, has not yet been explored. Therefore, this study focused on isolation, expansion and usage of patient-derived ECFC for recellularization purposes while comparing seeding efficiency of ECFC-mono- with ECFC-rMSC and ECFC-hMSC-coculture approaches on decellularized BCA-chips. Healthy rMSC were used due to easy availability and reported low immunogenicity [37]. Furthermore, a refined decellularization strategy was explored.

## Results

### Successful decellularization of bovine carotid arterial grafts

**Fig. 1:**
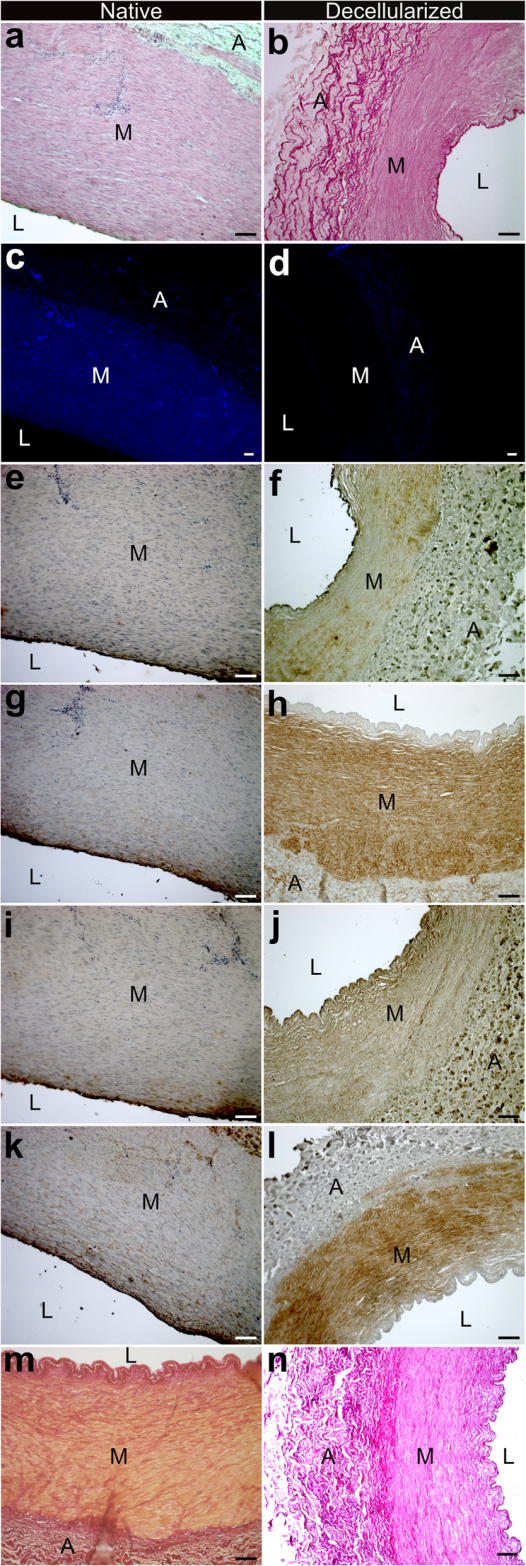
Native bovine carotid arteries (BCA, **a, c, e, g, i, k, m**) were compared to decellularized BCA (**b, d, f, h, j, l, n**). Following decellularization, complete removal of nuclei (**b, d**) was observed in H/E- (**a, b**) and DAPI (**c**, d) staining. Further histochemical characterization of ECM for collagen I (**e, f**), collagen IV (**g, h**), fibronectin (**i, j**), laminin (**k, l**) and Picrosirius red (**m, n**) showed successful retention of abundant ECM components. L, M and A indicate lumen, media and adventitia, respectively. Scale bar represents 100 μm

Histological evaluation of decellularized matrices showed complete removal of cellular material throughout the entire vessel wall in H&E- and DAPI-Staining. While typical arterial wall architecture remained intact, a thinner *tunica media* and *tunica adventitia* configuration was observed in decellularized samples **(**Fig. 1a-d). Immunohistochemical stainings for laminin, fibronectin, collagen-I and collagen-IV in addition to conventional picrosirius red staining were performed to characterize decellularized extracellular matrix and compared to native controls. Microscopic evaluation of ECM showed no loss of abundant ECM components in respective areas of typical appearance, however, the compressed configuration following cell removal resulted in an intensified appearance of ECM-elements, especially in the area of the *tunica media* (Fig. 1e-l). Picrosirius red staining showed collagen retention after decellularization with removal of cytoplasm (yellow, Fig. 1m, n).

### Biochemical analysis of decellularized matrices

**Fig. 2:**
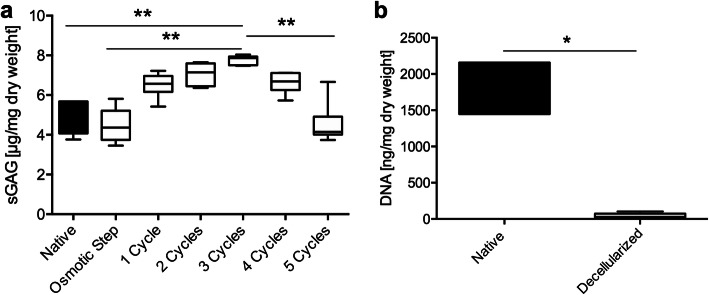
**a** The content of sulfated glycosaminoglycans (sGAG) per dry weight increased statistically significant following decellularization treatment after three consecutive detergent-enzymatic treatment cycles. Further incubation for four or five treatment cycles resulted in reduced sGAG content, which reached significance after five cycles. **b** DNA amount was reduced significantly after complete standard decellularization treatment (SDT) compared to native controls. * *p* < 0.05, ** *p* < 0.01

To evaluate content of sulfated glycosaminoglycans (sGAG) during decellularization process, sGAG content was measured in native tissue, after osmotic treatment and after multi-cyclic detergent-enzymatic treatment for up to five cycles (Fig. 2a). An increase of sGAG content was observed during decellularization, reaching a peak after three cycles of detergent-enzymatic treatment at 7.87 median (IQR 0.43) μg/mg dry weight. This was statistically significant (*p* = 0.007) when compared to native specimens (4.93 IQR 1.61 μg/mg dry weight, Fig. 2a). Further incubation with detergent-enzymatic treatment resulted in reduced sGAG content that reached significance after five consecutive cycles (4.15 IQR 0.90 μg/mg dry weight) when compared to maximum sGAG content after three detergent-enzymatic cycles (*p* = 0.0013, Fig. 2a). Therefore, standard decellularization treatment (SDT) was limited to detergent-enzymatic exposure for three consecutive cycles to prevent sGAG loss. To show effective decellularization, DNA content was measured after SDT and compared to native specimens. DNA content declined significantly during decellularization from 2157.00 (IQR 709.00) ng/mg dry weight in native BCAs to 30.00 (IQR 51.83) ng/mg dry weight after SDT (Fig. 2b).

### Mechanical properties of decellularized matrices

**Fig. 3:**
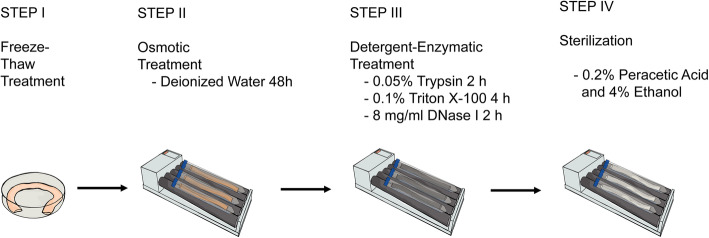
Graphic display of decellularization process. Decellularization treatment consisted of freeze-thaw treatment, osmotic treatment and multi-cyclic detergent-enzymatic treatment. To identify necessary cumulative incubation time for cell removal while preserving ECM-components, detergent-enzymatic treatment was performed in cycles, each consisting of trypsin-, Triton X-100- and DNase-treatment. Detergent-enzymatic decellularization cycles were continued for 5 cycles. Analytics showed complete cell removal in histology and significant DNA reduction while preserving sGAG content after three detergent-enzymatic decellularization cycles. SGAG wash out was observed starting at four or more detergent-enzymatic decellularization cycles, therefore standard decellularization treatment (SDT) was defined as freeze-thaw-treatment, osmotic treatment and three consecutive detergent-enzymatic decellularization cycles (cycles 4 and 5 are crossed out in figure). All decellularized specimens were sterilized in peracetic-acid-ethanol-solution prior to recellularization

**Fig. 4:**
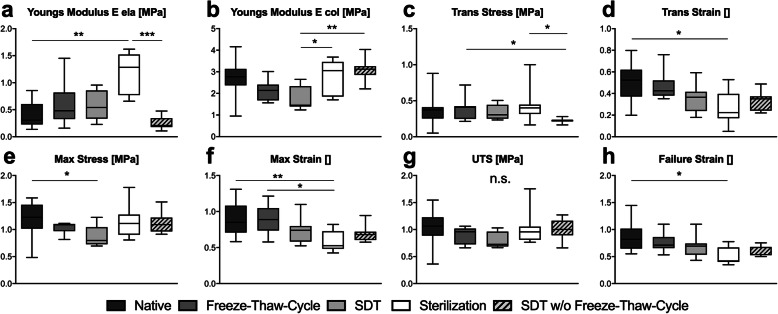
Mechanical properties of specimens during decellularization and sterilization process were determined and a moderate reduction of Young’s Modulus E col. (**b**), Max Stress (**e**) and UTS (**g**) was observed following freeze-thaw-cycle (*n* = 8) and standard decellularization treatment (SDT, *n* = 8) when compared to native controls (*n* = 12). Statistical significance was reached in the sterilization group with regards to Young’s Modulus E ela (**a**), Max Strain (**f**), Failure Strain (**h**) and Trans Strain (**d**). Final decellularized and sterilized BCAs (sterilization, *n* = 8) showed comparable results to native controls in regard to Youngs-Modulus E col. (**b**), Max Stress (**e**), UTS (**g**) and Trans Stress (**c**). SDT without freeze-thaw treatment (SDT w/o Freeze-Thaw-Cycle, *n* = 8) is shown for informatory purposes only but was not used to generate specimens intended for recellularization

To assess mechanical properties altered through the process of decellularization, native specimens (*n* = 12) were compared to samples acquired after freeze-thaw treatment (*n* = 8), SDT (*n* = 8), sterilization (*n* = 8) and SDT without Freeze-Thaw-Treatment (*n* = 9). The time point ‘sterilization’ represents the final vascular graft to be used for recellularization. SDT without Freeze-Thaw-Treatment is shown only for informatory purposes but was neither sterilized nor used for graft generation. Figure 3 better illustrates the decellularization and sterilization process. All samples were analyzed with regard to Young’s Modulus E during low (E ela, Fig. 4a) and high strain (E col., Fig. 4b), point of transition from E ela to E col. in regard to stress (Trans stress, Fig. 4c) and strain (Trans strain, Fig. 4d), maximum tolerated stress (Max stress, Fig. 4e) and strain (Max strain, Fig. 4f) and stress (UTC, Fig. 4g) and strain tolerated before rupture (Failure strain, Fig. 4h). Completely decellularized and sterilized samples showed comparable results to native controls by maintaining roughly 91% of initial stress resilience with regards to Max stress (native 1.23 IQR 0.44 MPa, sterilization 1.11 IQR 0.37 MPa, *p* > 1). Similar results were also observed for E col. (native 2.77 IQR 0.76 MPa, sterilization 3.05 IQR 1.61 MPa, p > 1), UTS (native 1.06 IQR 0.34 MPa, sterilization 0.96 IQR 0.24 N, *p* > 1) and Trans stress (native 0.37 IQR 0.16 MPa, sterilization 0.40 IQR 0.13 MPa, *p* > 1). Further characterization of mechanical behavior of samples acquired throughout the decellularization process revealed differing results. A limited decline of Max stress resilience was recorded after freeze-thaw treatment (1.09 IQR 0.13 MPa, 89% initial stress resilience) which reached statistical significance (*p* = 0.04) after SDT (Max stress 0.80 IQR 0.32 MPa, 65% initial stress resilience). Likewise, a decline of E col. could be seen in samples after freeze-thaw treatment and SDT (Freeze-Thaw-Cycle 2.14 IQR 0.73 MPa, SDT 1.43 IQR 0.94 MPa) which was followed by a statistically significant increase (*p* = 0.04) after sterilization (3.05 IQR 1.61 MPa) compared to SDT but comparable to native specimens. Max strain (native 0.85 IQR 0.37, sterilization 0.53 IQR 0.24 *p* < 0.01), Failure strain (native 1.00 IQR 0.27, sterilization 0.42 IQR 0.27 *p* = 0.01) and Trans strain (native 0.52 IQR 0.25, sterilization 0.22 IQR 0.23 *p* = 0.03) was reduced up to statistical significance after sterilization but showed no statistically relevant abbreviation to native controls for all other timepoints during graft generation for Max strain (Freeze-Thaw-Cycle 0.89 IQR 0.31, SDT 0.74 IQR 0.22), Failure strain (Freeze-Thaw-Cycle 0.71 IQR 0.20, SDT 0.70 IQR 0.21) and Trans strain (Freeze-Thaw-Cycle 0.43 IQR 0.14, SDT 0.37 IQR 0.18). While a reduction of UTS was observed for all groups (Freeze-Thaw-Cycle 0.96 IQR 0.29, SDT 0.73 IQR 0.28), it did not reach statistical significance. Trans stress did not show any statistically significant changes throughout graft generation process (Trans stress 0.30 IQR 0.19 MPa). E ela, however, showed an increase during graft generation that reached statistical significance after sterilization (native 0.31 IQR 0.34 MPa, Freeze-Thaw-Cycle 0.48 IQR 0.49 MPa, SDT 0.54 IQR 0.52 MPa, sterilization 1.28 IQR 0.75 MPa *p* = 0.008). SDT without Freeze-Thaw-Cycle showed significantly altered mechanical test results compared to sterilization (E ela 0.21 IQR 0.17 MPa, *p* < 0.001, Trans stress 0.23 IQR 0.02 MPa, *p* = 0.016), SDT (E col. 3.12 IQR 0.40 MPa, *p* = 0.004) and Freeze-Thaw-Cycle (Trans stress 0.41 IQR 0.18 MPa, *p* = 0.049) while alle other measurements remained comparable (UTS 1.00 IQR 0.27 MPa, Failure strain 0.67 IQR 0.15, Trans strain 0.35 IQR 0.13, Max stress 1.09 IQR 0.26 MPa, Max strain 0.68 IQR 0.12). Complete data is available as a supplemental file.

### Patient characteristics

In total, ECFC were isolated from 10 patients in this study (Table 1). The patients were equally male (50%) and female (50%), and the median age of patients at the time of ECFC-isolation was 62 years. All patients underwent surgery prior to ECFC-isolation. Oncological, general and endocrinological surgery was performed in 40, 40 and 20% of the patients, respectively. Most common comorbidities were arterial hypertension (80%), renal failure (40%) and diabetes (30%). None of the patients were active smokers.

**Table 1:** Characteristics of donors of endothelial colony forming cells

Characteristics	***N*** = 10
Male sex, %	50
Median age at isolation (range), years	62 (41–83)
Non-Smoking, %	100
Post-surgical, %	100
Co-morbidities, %	
Arterial hypertonia	80
Diabetes	30
Renal failure	40
Coronary heart disease	10
Admission by surgical departments, %	
Oncological surgery	40
General surgery	40
Endocrinological surgery	20

### Isolation of endothelial colony forming cells

**Fig. 5:**
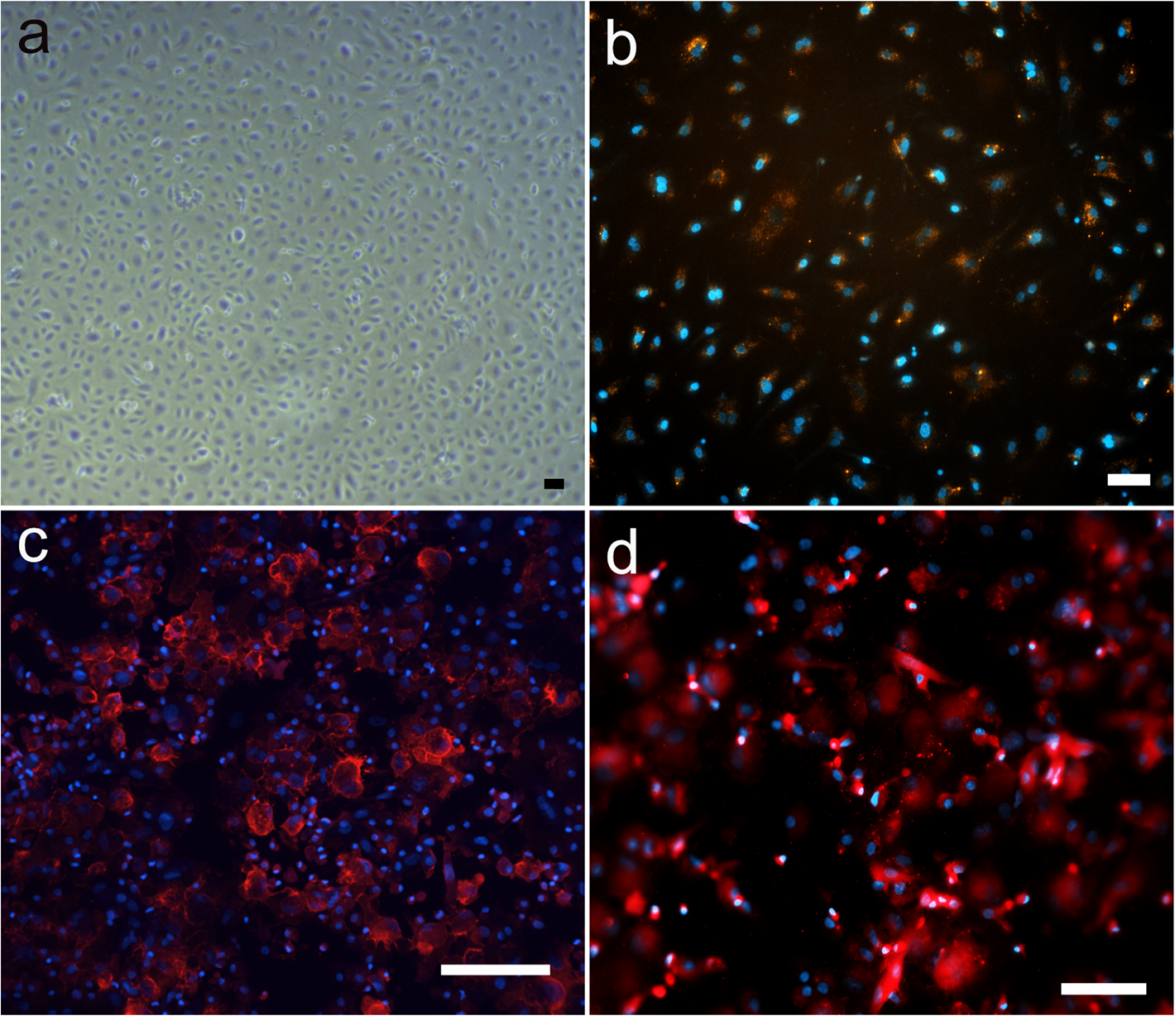
Inverted brightfield and immunofluorescence microscopy of endothelial colony forming cells (ECFC). Inverted brightfield microscopy showed cobblestone-like appearance (**a**). Fluorescent microscopic images of ECFC show nuclei in blue (DAPI; **b,c,d**) in colocalization with DiI-acLDL (orange, **b**), CD31 (red, **c**) and von Willebrand factor (red, **d**). Scale bars represent 100 μm (**b,c,d**) or 200 μm (**a**) respectively

Cell-Isolation via peripheral blood draw from hospitalized patients was successful in 8 of 10 patients. Cell culture contamination led to the exclusion of two cell-isolations, leaving a total of 8 cell-isolations used for further experiments. All included patients were suffering from either diabetes, hypertension, chronic renal failure or a combination thereof (Table 1). Cell cultures were screened daily until cobblestone-like colonies emerged at a mean of 10-12d (Fig. 5). Rapid doubling times followed colony appearance. Acetylated low-density lipoprotein (acLDL) uptake was observed in ECFC cultures incubated with DiI-acLDL by means of colocalization of DAPI- and surrounding DiI-signal (Fig. 5b). Immunofluorescence staining for CD31 (Fig. 5c) and vWF (Fig. 5d) was performed on CytoSpin specimens. Due to the manner of CytoSpin-preparation, no results in regard to cell-cell interaction or cell-colony formation could be obtained. Cells generally appeared dense and layered and stained positive on cell surface area for CD31 or within vesicle-like structures consistent with Weibel-Palade-Bodies for vWF.

### Seeding of hECFC on ECM-chips

**Fig. 6:**
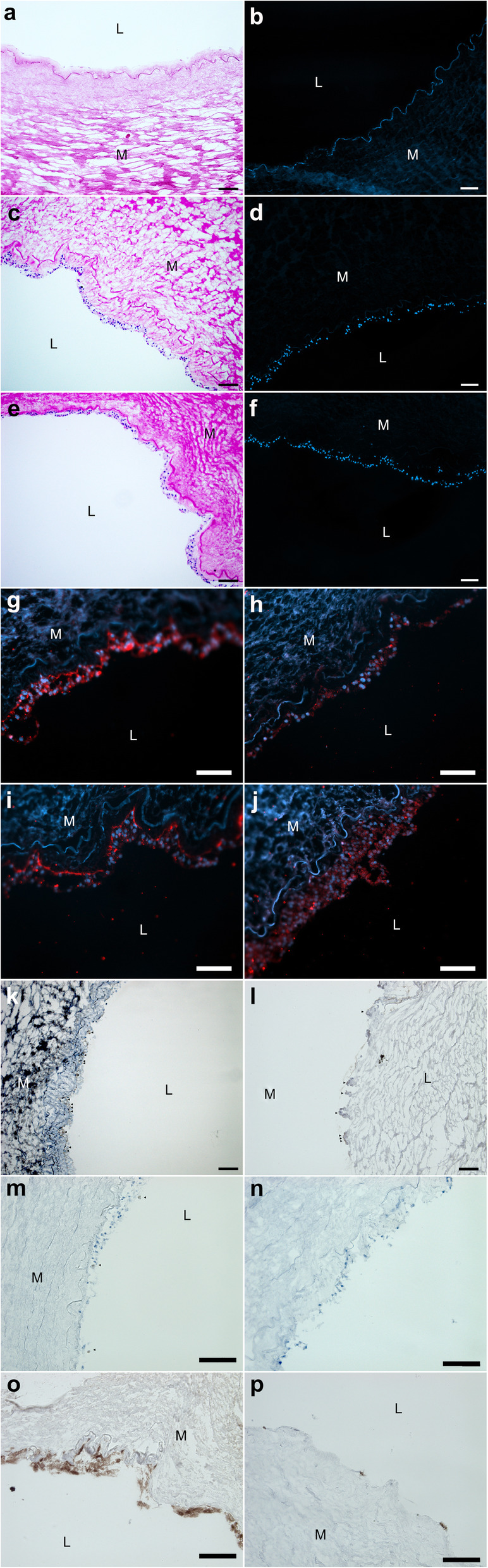
Inverted brightfield and immunofluorescence microscopy of recellularized extracellular matrix (ECM) chips. ECM-chips were seeded with endothelialcolony forming cells (ECFC) in monoculture (**a**, **b**) or in coculture with rat (rMSC, **c-k**, **m**, **n**) or human mesenchymal stromal cells (hMSC, l, **o**, **p**) andincubated for 14 (**a-d**, **g**, **h**, **m**) or 23 days (**e**, **f**, **i**, **j**, **k**, **l**, **n-p**). H&E (**a**, **c**, **e**), DAPI (**b**, **d**, **f**), vWF (**g**, **i**) and CD31 (**h**, **j**, **o**) staining revealed confluent cellattachment on luminal ECM-chip side. A trend towards layered cell growth was observed in samples incubated for 23 days (**e**, **f**, **i**, **j**). CD90 (**k**, **l**, arrows),HNA (**m**, **n**, **p**) staining was observed. Scale bar represents 100 μm

Decellularized ECM-chips were seeded with human endothelial colony forming cells (hECFC). Recellularized ECM-chips were examined for cell growth using brightfield and fluorescence microscopy. DAPI- and H/E-staining of ECM-chips recellularized with hECFC in monoculture for 12 days showed limited to no seeding efficiency. Solitary cells or cell residue on luminal ECM-chip-side could be found in only one of nine recellularized samples (*n* = 9) in H&E staining (Fig. 6a). We were unable to confirm any cell nuclei in DAPI-staining (Fig. 6b).

### Seeding of co-cultured hECFC with rMSC on ECM-chips

Easily available rat mesenchymal stromal cells (rMSC) were used for co-culture applications. Matrices seeded with hECFC in co-culture with rMSC showed confluent cell attachment to luminal ECM-chips on all recellularized samples. Cell growth remained exclusively along the luminal chip side and no infiltration of cells into the tunica media or adventitia was observed. This was true for both 14d (*n* = 8, Fig. 6c-d) and 23d (*n* = 5, Fig. 6e-f) culture periods. Longer incubation times favored layered cell growth, as this was observed in samples incubated for 23 days more frequently and more distinctly than after 14 days of culture. Cell nuclei appeared rounded in both H&E- and DAPI-staining for all co-culture approaches. To define the cell origin of seeded cells, immunofluorescence staining was performed for CD31 and vWF as well as CD90 and HNA. Impressive colocalized signal of both CD31 and vWF in regard to DAPI-Signal was observed in confluent cell layers (Fig. 6g-j). The positive immunofluorescent signal appeared in the respective cell areas. Notably, subendothelial vWF-signal, consistent with the typical endothelial cell vWF-storage location, was observed in both culture periods. Furthermore, vWF-Signal was intensified after 23 days of culture (Fig. 6g,i). Scattered CD90-positive staining showed presence of MSC after 23 days for co-culture with rat or human MSC (Fig. 6k [rat], Fig. 6m [human], arrows) while negative control showed no positive signal (Suppl.). However, HNA staining indicated that most of CD 31 and VWF-positive cells originated from rMSC. Only a few HNA positive cells could be found after 14 (Fig. 6m) and none after 23 days (Fig. 6n) of culture.

### Seeding of co-cultured hECFC with hMSC on ECM-chips

Human mesenchymal stromal cells (hMSC) were also used for co-culture applications. Matrices seeded with hECFC in co-culture with hMSC showed partially confluent cell attachment to luminal ECM-chips after 23 days of culture (Fig. 6l). Immunohistological staining was performed for CD31 (Fig. 6o) and CD90 (Fig. 6l). Again, CD31 positive staining was observed. CD90-positive staining showed presence of hMSC after 23 days (Fig. 6l). Few HNA positive cells were found after 23 days of culture (Fig. 6p).

## Discussion

In this study we evaluate the capability of ECFC acquired through peripheral blood draw from hospitalized patients to reendothelialize decellularized BCA chips when applied in co-culture with rMSC and hMSC. The results may help to better estimate seeding capabilities of ECFC derived from the intended target group for reendothelialization.

Opposite to autologous vessels, TEVG are not a scarce material and could be implanted at an early disease stage. Ideally, artificial surface reaction through current non-biological devices could be avoided and, as a result, TEVG would be implanted in tissue with fewer inflammatory alterations. Constructive remodeling [46] and further improved patency rates could be obtained by this strategy. In order to acquire readily available and therefore transplantable scaffolds for recellularization, discarded BCAs were decellularized using a combination of decellularization techniques for gentle yet effective cell removal. To prevent inferior mechanical properties [47] and reduced recellularization capabilities through known drastic ECM-modification [48] by sodium dodecyl sulphate (SDS), the main reasons for both early and late graft failure, we modified the clinically successfully applied SDS-free protocol by Olausson et al. [49] Also, a SDS-free approach might evade removal and alteration of base membrane proteins and both less confluent and atypical cell growth [50]. Cell lysis was therefore initiated through a freeze-thaw cycle and incubation with deionized water [51]. Further cell lysis and cell debris removal was obtained by a combination of incubation with trypsin, Triton X-100 and DNase. While non-ionic detergent Triton X-100 is known to affect sGAG modification mildly [50] and was used in lower concentrations than comparable protocols [49, 50], sGAG content was significantly reduced after five detergent-enzymatic treatment cycles. Successful decellularization through sufficient DNA reduction to < 50 ng/mg dry weight and absence of cell nuclei in respective histology [51] was achieved after three detergent-enzymatic cycles, a prerequisite to avoid chronic inflammation and allow constructive remodeling when using scaffolds of xenogenic origin [52]. We limited the exposure of specimens accordingly.

Evaluating mechanical properties at defined time points we saw varying mechanical properties throughout our proposed decellularization protocol. An expected but statistically insignificant decline of strength retention and Youngs Modulus E col. following freeze-thaw treatment may be explained by minor ECM disruption of mainly collagen fibers [51]. A preservation of mechanical properties at statistically significant lower values following SDT but mostly comparable results to native controls following additional peracetic acid (PAA) sterilization process was observed. PAA sterilization has been linked with altered mechanical properties due to oxygen free radical reactivity and crosslinking leading to both tissue specific biaxial strength increase (submucosa) and decrease (bladder) [53, 54] but was used as PAA treated ECM showed unaltered or improved reseeding capabilities [55, 56] before. Considering preservation of key mechanical components and sGAG content as well as no detectable cell growth beyond the luminal graft side indicating retained ECM characteristics., the proposed decellularization and sterilization process seems to be favorable. In summary the decellularized and sterilized graft, suitable for recellularization, closely mimics native mechanical characteristics while altered mechanical characteristics may be observed during the decellularization process.

While using ECFC obtained from healthy individuals for recellularization approaches has become common practice and is performed successfully, it does not address the goal to develop autologous treatment options for the intended high-risk cardiovascular target group sufficiently. Krawiec et al. [35] were the first to identify and use adipose-derived stromal cells obtained from a cardiovascular risk group as a potential cell source for TEVG generation while expressing the need to evaluate additional cell groups used for tissue engineering. Altered function and reduced ECFC numbers have not only been observed in aging patients, but also in patients suffering from diabetes, hypertension and chronic renal failure, all being risk factors for PAD [57, 58] and indicating a key role of ECFC in cardiovascular disease [40–42]. We therefore evaluated both, the isolation and seeding efficiency of ECFC from high-risk donor populations in this study. Our results demonstrate that patient-derived ECFC can be isolated through peripheral blood draw and expanded successfully ex vivo.

To answer the vital question whether patient-derived ECFC may grow on decellularized xenogenic matrices, BCA-chips were recellularized. In contrast to published data stating successful recellularization using healthy ECFC [25, 54–56], decellularized BCA-chips seeded with patient-derived ECFC showed unsatisfactory seeding efficiency. As improved vasculogenic properties of peripheral blood ECFC co-implanted with MSC have been reported, we hypothesized that especially impaired patient-derived ECFC are dependent on perivascular support [29, 59]. ECFC and MSC are known to interact via both, cell-cell-contacts and paracrine mechanisms. We therefore assumed co-implantation could help both cell types to maintain their respective cell function and enable remodeling as well as anastomosis healing upon implantation in vivo and exposure to patient-specific serum. Additionally, MSC have shown to be both, immune evasive and immunosuppressive and can therefore create a low inflammatory environment [60], suitable for constructive remodeling [46]. To explore our hypothesis we used both rat bone-marrow MSC (rMSC) due to easy obtainability and reported low immunogenicity [61] as well as human umbilical cord MSC (hMSC). Published research showed both positive and limiting effects in regard to cell proliferation and survival when comparing allogenic and xenogenic co-culture of ECFC and MSC [62–64]. However, so far only a limited amount of data has been collected and may lead to a distortion of the results in both a positive and negative emphasis.

Unexpectedly, by using a xenogenic co-seeding approach we were able to show that while CD31 and vWF expression in EC typical patterns occurred, these cells did not stain positive for HNA. Therefore, rMSC seemed to express both, CD31 and vWF, and outnumbered ECFC after 14 and 23 days of culture. Under static conditions, ECFC did not survive in great numbers but may have rather supported the differentiation of MSC towards endothelial fate. Co-culture of hMSC and ECFC under similar seeding and cell-culture conditions showed inferior partial reendothelialization compared to rMSC co-culture. Differing seeding-efficiency and cell-survival may be attributed to either heterogeneous ECFC or varying MSC-sources or both. Additionally, different culture-mediums for MSC could have influenced cell behavior. In summary, comparable to unsatisfactory results in regard to seeding capability of ECFC isolated from high-cardiovascular-risk patients reported previously [65], we saw low seeding capability in co-seeding experiments with MSC [29].

Our findings serve as a reminder that sick cells from patients might provide different characteristics from young and healthy cells. So far, we have only seeded ECM-chips in a two-dimensional cell-culture approach of TEVG-chips of only 0.5 cm^2^. It remains possible that confluent coverage and function of ECFC and MSC will remain intact and show proper function upon exposure to in vivo conditions. Also, regular function of patient-derived MSC is still unclear and should be investigated through rigorous functional testing as others have shown intimal hyperplasia leading to graft-failure upon implantation in vivo [66]. While we have shown comparable mechanical attributes for decellularized scaffolds, in depth biomechanical analysis especially for smaller loads without pre-strain and multiple directions are still to be addressed, preferably using direct testing methods to omit overestimating mechanical characteristics [67].

In order to facilitate the translation of our collected data into clinical applications, additional research is required. In a first step the application of our findings should be evaluated to create a bypass graft from both human ECFC and human MSC of reasonable lengths under physiological flow and pressure conditions, possibly using bioreactors. This would also address the vital question if sick human MSC can be as beneficial as healthy rat MSC. This could be addressed in an animal-free cell and culture medium model using realistically achievable cell counts for seeding. To develop a patient-derived autologous TEVG, its reendothelialization in cylindrical form, maturation and implantation would be the logical next step.

## Conclusion

In this proof-of-concept study we are the first to show the inability of patient-derived ECFC, isolated from patients who display a corresponding deposition due to their age and cardiovascular risk factors for the development of PAD, to form a confluent endothelial layer on decellularized bovine carotid arteries. Successful endothelialization was achieved upon co-culture of patient-derived ECFC with healthy rat MSC and partial endothelialization was observed for the co-culture of patient-derived ECFC with healthy human MSC. Under static conditions, MSC formed a confluent cell layer while expressing typical endothelial cell markers. Furthermore, we show a refined decellularization strategy for widely available BCAs to retain key mechanic and ECM characteristics through an SDS-free approach to be used as small-diameter bypass for patients with PAD.

## Methods

All methods were carried out in accordance with relevant guidelines and regulations. Special permission was obtained for all experimental procedures with local committees and institutions where applicable.

### Vessel harvesting

BCAs were obtained from a slaughterhouse and transported in cooled phosphate buffered saline (PBS) pH 7.4 (PBS, Biochrom, Berlin, Germany) to the laboratory facilities of the Department of Surgery, where surplus tissue was removed. Vessels were washed with PBS and stored frozen at − 80 °C until further use.

### Preparation of Acellular matrices

The standard decellularization treatment (SDT) consisted of three steps as visualized in Fig. 1:

- *Step 1 Freeze-Thaw-Treatment:* BCA were frozen at − 80 °C for > 24 h and then thawed to 5 °C.
- *Step 2 Osmotic Treatment:* BCA were rinsed with deionized water for 48 h on an elliptical tube roller mixer (RM 5, Glaswarenfabrik Karl Hecht, Sondheim, Germany).
- *Step 3 Multi-Cyclic Detergent-Enzymatic Treatment:* Multi-cyclic detergent-enzymatic treatment was performed daily for three consecutive days on a roller mixer at 37 °C. BCA were treated each day with 0.05% Trypsin (Sigma-Aldrich, St. Louis, MO, USA) for 2 h, 0.1% Triton-X 100 (Roth, Karlsruhe, Germany) for 4 h, both diluted in 0.05% EGTA (Sigma-Aldrich), and 8 mg/mL DNase-I (Roche Diagnostics, Risch, Switzerland) for 2 h. DNase-I was dissolved in DNase reaction buffer containing 10 mM Tris-HCl, 2.5 mM MgCl2 and 0.5 mM CaCl2 at pH 7.6 at room temperature (RT). Vessels were washed with PBS between treatment-steps. BCAs were stored in PBS solution containing 1% Penicillin/Streptomycin (Biochrom) at 5 °C. *Step 3* was prolonged to 5 days to evaluate sGAG content.

### Biochemical analysis of Acellular matrices

Tissue samples of native and decellularized BCA were homogenized and lyophilized. For all biochemical analysis, processed specimens were compared to native controls using 10 mg dry weight samples.

### sGAG-content of Acellular matrices

Sulfated glycosaminoglycans (sGAG) content was measured following osmotic treatment and after each Multi-Cyclic Detergent-Enzymatic Treatment for 5 days using the previously published protocol by Farndale et al. [68] Native and decellularized tissue samples as well as Chondroitin-4-Sulphate (0–200 μg/mL, Roth) to acquire a standard curve, were incubated in papain-containing-buffer, mixed with 1,9-Dimethylmethylene blue (DMMB) at equal parts and measured at a wavelength of 525 nm using the NanoDrop 2000C spectrophotometer (Thermo Fisher Scientific, Waltham, MA, USA).

### DNA-content of Acellular matrices

DNA-Content was obtained using DNeasy Blood & Tissue Kit (Qiagen, Venlo, Netherlands) according to the manufacturer’s instructions. Samples were measured using the NanoDrop 2000C.

### Mechanical analysis of Acellular matrices

Acellular Matrices were cut into 5 mm wide rings with 5 mm diameters. The thickness was measured while using a digital gage (accuracy: ± 0.1 mm; Mitutoyo, Andover, UK). Subsequently the ring specimens were mounted on two custom made wire hooks and finally subjected to low strain rate uniaxial tensile loading to failure testing by means of an Bose tensile machine device (BOSE ElectroForce LM1, Bose Corporation, Eden Prairie, MN, USA). Specimens were manually prestrained up to approximately 1 N and subsequently loaded until failure at 0.166 mm/s, while applied force and displacement was acquired at a 100 Hz sample rate. Further data analysis was executed using a routine written in MATLAB (The MathWorks, Inc., Natick, MA, USA) converting force (F, N) and displacement (d, mm) to engineering stress (σ, MPa) and strain (ε, adimensional). The stress-strain curve of each sample was plotted, and the following parameters were calculated: elastic phase modulus (E ela, MPa) and collagen phase modulus (E col., MPa), representing Young’s modulus under low and high strain determined as linear part of the stress-strain-curve, respectively. Ultimate tensile strength (UTS, MPa), indicating at which stage the sample failed and the corresponding stress-strain-curve exhibits the first drop in load, furthermore the maximal stress (Max stress, MPa) as the highest stress experienced. Max strain, Failure strain and Trans strain are defined accordingly (adimensional). Finally, the point at which the slope of E ela and E col. meet (see Suppl.) defines Trans strain and the corresponding point on the stress-strain-curve provides Transition stress (MPa). Native (*n* = 12) and specimens having undergone freeze-thaw-Ttreatment, SDT and sterilization (*n* = 8 each) as well as SDT without freeze-thaw-treetment (*n* = 9) were compared.

### Histological analysis of Acellular matrices

Native vessels and decellularized samples were fixed with 4% paraformaldehyde (PFA, Roth), dehydrated, paraffinized and cut into 7 μm thick sections. Sections were stained with hematoxylin (ITW Reagents, Darmstadt, Germany) and eosin (Morphisto, Frankfurt a.M., Germany) or DAPI (Sigma-Aldrich) to verify the absence of cellular elements and DNA residue. Immunohistochemical stainings for Laminin, Fibronectin, Collagen-I and Collagen-IV as well as conventional picrosirius red staining for collagen were performed to evaluate extracellular matrix components. Table 2 states primary and secondary antibodies used for histological analysis. Following deparaffinization and rehydration, all immunohistochemical samples underwent 3% peroxidase block and 0.1 M citrate buffer pH 6 antigen retrieval. Respective primary antibodies for fibronectin and collagen I staining were incubated for 2 h at 37 °C. Designated samples for laminin and collagen IV staining were blocked with 3% goat serum and incubated with primary (1 h) and secondary antibody (30 min, goat-anti-rabbit, ab6721, abcam) at 37 °C. All remaining steps were performed using LSAB2 Kit (K0675, Agilent Technologies, Santa Clara, CA, USA).

**Table 2:** Overview of antibodies used

Target	Host	Dilution	Cat.-No.	Manufacturer
**Primary Antibodies**				
Collagen-I	Mouse	1:400	H00001278-M03	Abnova, Taipei, Taiwan
Collagen-IV	Rabbit	1:400	Ab6586	abcam, Cambridge, UK
Fibronectin	Rabbit	1:400	Ab23751	abcam, Cambridge, UK
Laminin	Rabbit	1:50	Ab11575	abcam, Cambridge, UK
vWF	Rabbit	1:400	Ab6994	abcam, Cambridge, UK
CD31	Rabbit	1:100	Bs-0195R	Bioss Antibodies Inc. Woburn, USA
CD90	Rabbit	1:10	Ab92574	abcam, Cambridge, UK
HNA	Mouse	1:200	Ab191181	abcam, Cambridge, UK
**Secondary Antibodies**				
Rabbit	Goat	1:400	Ab6721	abcam, Cambridge, UK
Mouse	Donkey	1:400	715–035-150	Dianova, Hamburg, Germany
Rabbit	Goat	1:400	Ab150080	abcam, Cambridge, UK

### Isolation of Endothelial Colony Forming Cells (ECFC)

ECFC were isolated similarly to previously published protocols [69, 70]. Following approval of the ethics committee of the Charité – Universitätsmedizin Berlin (EA1/256/14) and in accordance with the declaration of Helsinki, peripheral blood was obtained from hospitalized patients after informed consent. Blood samples were heparinized and treated separately for each patient. Mononuclear cells were isolated using Biocoll (Biochrom) density gradient centrifugation. Mononuclear cell suspension was subsequently plated on fibronectin-gelatin-coating (Fibronectin 0.005 mg/ml, Merck; Gelatin 0.2 mg/ml, Sigma-Aldrich) and incubated at 37 °C and 5% CO_2_ for 4 days with EBM-2 medium (Lonza, Basel, Switzerland) supplemented with EGM-2 SingleQuots (Lonza), 18% Fetal Bovine Serum (FBS, Merck) and 1% Penicillin-Streptomycin (P/S, EGM-2) to reach a final concentration of 20% FBS in EGM-2.). Afterwards, all non-adherent cells were washed off with PBS, new EGM-2 medium was added, changed daily for 1 week and then every other day. ECFC were expanded by replating at 80–90% confluence.

### Characterization of isolated ECFC

Cell cultures were assessed daily for cell growth, colony formation and absence of contamination with an Axiovert 40 CFL microscope (Carl Zeiss AG, Oberkochen, Germany). Isolated ECFC were fixated on microscope slides using the Cytospin (Cytospin 4, Thermo Fisher Scientific) procedure: Cells were trypsinized, aliquoted, centrifuged onto slides and fixated with Merckofix (Merck). Slides were then washed, incubated with protein block (Agilent Technologies) for 45 min (CD31) or PBS containing 1% bovine serum albumin (BSA, Agilent Technologies) and 0.2% Triton X-100 for 10 min (vWF) at RT. Antibodies were diluted in PBS containing 1% BSA and 1% goat serum. Samples were incubated with primary antibodies for 1 h at 37 °C (CD31) or overnight at 4 °C (vWF) before being incubated with the secondary antibody for 45 min at 37 °C. Afterwards, the cells were washed, counterstained with DAPI and mounted with Aquatex (Merck). Isolated ECFC were incubated with EGM-2 Medium containing 10 μg DiI-ac-LDL (Merck) for 4 h at 37 °C and 5% CO_2_. The cells were then washed, counterstained with DAPI, fixated with 4% PFA and microscopically assessed.

### rMSC isolation

Rat mesenchymal stromal cells (rMSC) were isolated from rat femur bone marrow according to a modified protocol after Soleimani and Nadri [71]. Bones serving as cell sources for rMSC Isolation had been discarded hind limbs from other animal experiments. All initial animal work was performed in accordance with local law and approved by the State Office of Health and Local Affairs (Landesamt für Gesundheit und Soziales, LAGeSo, Berlin, Germany; Reg. No. T 0139/13, T 0301/17 and O 0365/11). Briefly, following femur explantation, bones were sterilized in 70% Ethanol and manually snapped in half under sterile conditions. The intramedullary canal was rinsed with RPMI-Medium (Thermo-Fischer Scientific) supplemented with 10% FCS and 1% P/S, (RPMI), the cell-containing medium was transferred to a cell culture flask (Corning Inc.) and incubated at 37 °C 5% CO_2_. After 4 days, the cell culture flasks were rinsed, inspected for adherent cells and fresh RPMI medium was added. rMSC were replated at 70% confluence.

### Recellularization of acellular BCA matrices with hECFC or hECFC in coculture with rMSC or hMSC

All steps were performed under sterile conditions. Decellularized matrices were sterilized by incubation for 6 h with 0.2% Peracetic Acid (PAA) and 4% Ethanol (EtOH) followed by washing steps in sterile PBS until the pH was neutralized. The matrices were then cut to 0.5 cm^2^ pieces and equilibrated in EGM-2 in 12-well plates overnight.

ECFC derived from high cardiovascular risk donors were used for all recellularization experiments. Expanded hECFC were trypsinized, washed and resuspended in EGM-2 before seeding on the luminal side of matrices in 80 μl EGM-2 containing 3.6 × 10^5^ hECFC. Seeding medium consisting of equal parts of EGM-2 medium and M-199 medium (rMSC) containing 10% FBS and 1% P/S (seeding medium) or ATCC PCS-500-030 Medium supplemented with ATCC PCS-500-040 (hMSC) was prepared. Expanded hECFC and rMSC or human MSC (hMSC, human umbilical cord-derived mesenchymal stem cells, ATCC) were trypsinized, washed and resuspended in seeding medium. Approximately 80 μl of cell suspension containing 5.5 × 10^5^ ECFC and 4.2 × 10^4^ rMSC or hMSC was added to the luminal side of each matrix followed by 1 h incubation at 37 °C 5% CO_2_. Subsequently, 4 mL of seeding medium was added and seeded matrices were incubated for 72 h at 37 °C and 5% CO_2_. After 72 h matrices were transferred carefully to new 12 well plates. Incubation continued for 12 (*n* = 8) days in monoculture and 14 (*n* = 6) or 23 (rat (*n* = 5) or human (*n* = 6) MSC) days in co-culture experiments.

### Analysis of Recellularized matrices

Native vessels and recellularized samples were embedded in Tissue Freezing Medium (Leica), snap-frozen and stored at − 80 °C. Cryostat sections (7 μm, Thermo Fisher Scientific) were prepared, fixated in cold acetone (Roth), air-dried and washed with PBS. Sections were stained with H&E and DAPI. To prove endothelial origin, CD31- and vWF-staining was performed. Following incubation in boiling 0.01 M Target Retrieval Solution pH 6 (Agilent Technologies) for 20 min, specimens were treated with blocking buffer containing 3% goat serum and 0.3% Triton X-100 for 45 min at RT. Antibodies were diluted in buffer containing 1% BSA, 1% goat serum and 0.3% Triton X-100. Primary antibodies were then applied to tissue samples and incubated at 4 °C overnight. The secondary antibody (1:400, ab150080, abcam) was applied to tissue samples following washing for 45 min at 37 °C. Specimens were then washed, counterstained with DAPI and coverslipped using Aquatex (Sigma-Aldrich). To show cell distribution of human- and MSC-origin CD90 and human nuclear antigen (HNA) histochemical staining was performed. Epitope retrieval by using the pressure cooker method for 5 min while specimens were placed in boiling TRIS/EDTA-buffer and peroxidase blocking was performed for CD90 staining. Blocking was performed using protein block for HNA. Primary antibody (1:20, ab92574; 1:200, ab191181) was then applied and incubated over night at room-temperature. The secondary antibody (1:400, ab6721; 1:400, 715–035-150) was applied to tissue samples following washing for 45 min at 37 °C (CD90) or 30 min at RT (HNA). All remaining steps were performed using LSAB2 Kit (K0675, Agilent Technologies, Santa Clara, CA, USA). Microscopic evaluation was performed using Observer Z1 microscope (Carl Zeiss AG) or EVOS FL Auto microscope (ThermoFisher Scientific, Fig. 5c).

### Statistical analysis

All calculations were performed using GraphPad Prism version 6.04 for Mac, GraphPad Software, La Jolla, CA, USA. Data was tested for normality using Shapiro-Wilk test. Statistical comparison was performed by either Friedman test (sGAG-content), Wilcoxon test (DNA-content) or Kruskal-Wallis test (mechanical data). Data is presented as median and interquartile range (IQR). *p*-Values below 0.05 were considered statistically significant. All graphs in this study show medians with the respective IQR.

## Supplementary Information

**Additional file 1.** Shows complete data of the mechanical experiment including stress-strain curves and negative control images for histological staining.

## Data Availability

The datasets used and/or analyzed during the current study are available from the corresponding author on reasonable request.
