# Supplementary material for: In vitro recellularization of decellularized bovine carotid arteries using human endothelial colony forming cells
Source: J Biol Eng. 2021 Apr 21;15:15. doi: 10.1186/s13036-021-00266-5 (PMC8059238; doi:10.1186/s13036-021-00266-5)

In vitro Recellularization of Decellularized Bovine Carotid Arteries using human Endothelial Colony Forming Cells

Nicolai Seiffert[1,2], Peter Tang[1], Eriselda Keshi[1], Anja Reutzel-Selke[1], Simon Moosburner[1], Hannah Everwien[1,5], Dag Wulsten[3], Hendrik Napierala[1], Johann Pratschke[1], Igor M. Sauer[1,*], Karl H. Hillebrandt[1,6,7], Benjamin Struecker[4,6,7]

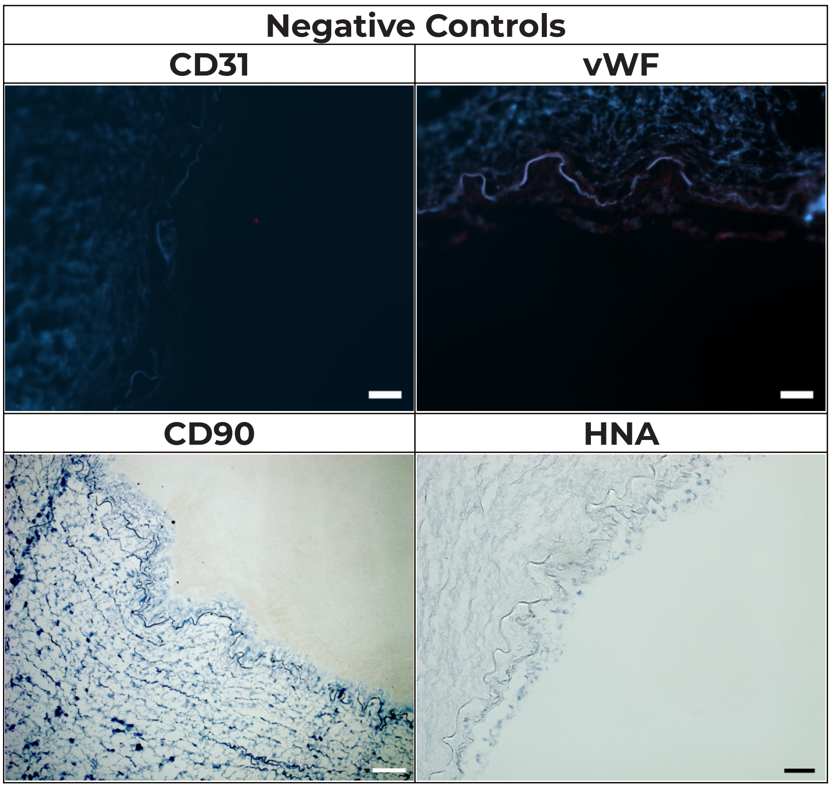

Sample	E ela [MPa]	E coll [MPa]	UTS [MPa]	Failure strain []	Transstress [MPa]	Transstrain []	Max stress [MPa]	Max strain []
**Native**								
25% Percentile	0,2282	2,372	0,8879	0,8942	0,2536	0,372	1,015	0,7083
**Median**	**0,3051**	**2,768**	**1,063**	**1,002**	**0,3721**	**0,5239**	**1,228**	**0,8506**
75% Percentile	0,6067	3,135	1,228	1,164	0,4109	0,6205	1,459	1,082
**IQR**	**0,3785**	**0,763**	**0,3401**	**0,2698**	**0,1573**	**0,2485**	**0,444**	**0,3737**
**Freeze-Thaw-Cycle**								
25% Percentile	0,3268	1,676	0,7264	0,6564	0,2478	0,3802	0,9714	0,737
**Median**	**0,4813**	**2,139**	**0,9589**	**0,7131**	**0,4081**	**0,4251**	**1,089**	**0,8887**
75% Percentile	0,8185	2,404	1,019	0,8601	0,4242	0,5214	1,105	1,048
**IQR**	**0,4917**	**0,728**	**0,2926**	**0,2037**	**0,1764**	**0,1412**	**0,1336**	**0,311**
**SDT**								
25% Percentile	0,3337	1,393	0,6868	0,535	0,2506	0,239	0,7283	0,583
**Median**	**0,5404**	**1,453**	**0,7278**	**0,6935**	**0,3039**	**0,3656**	**0,7984**	**0,7401**
75% Percentile	0,8583	2,336	0,962	0,7419	0,4432	0,4165	1,045	0,7989
**IQR**	**0,5246**	**0,943**	**0,2752**	**0,2069**	**0,1926**	**0,1775**	**0,3167**	**0,2159**
**SDT +Sterilization**								
25% Percentile	0,7711	1,847	0,8111	0,4013	0,3152	0,1709	0,9043	0,4827
**Median**	**1,284**	**3,052**	**0,9576**	**0,4188**	**0,4**	**0,2234**	**1,113**	**0,5255**
75% Percentile	1,522	3,458	1,054	0,6691	0,4452	0,3969	1,278	0,7272
**IQR**	**0,7509**	**1,611**	**0,2429**	**0,2678**	**0,13**	**0,226**	**0,3737**	**0,2445**
**SDT w/o Freeze-Thaw-Cycle**								
25% Percentile	0,1787	2,857	0,8942	0,5257	0,2124	0,2411	0,964	0,6039
**Median**	**0,2093**	**3,121**	**1,002**	**0,6699**	**0,2298**	**0,3507**	**1,093**	**0,6802**
75% Percentile	0,3439	3,262	1,164	0,6781	0,233	0,3734	1,223	0,7201
**IQR**	**0,1652**	**0,405**	**0,2698**	**0,1524**	**0,0206**	**0,1323**	**0,259**	**0,1162**

In vitro Recellularization of Decellularized Bovine Carotid Arteries using human Endothelial Colony Forming Cells: One Step Closer to a huminized Bypass Graft

Nicolai Seiffert[1,2], Peter Tang[1], Eriselda Keshi[1], Anja Reutzel-Selke[1], Simon Moosburner[1], Hannah Everwien[1,5], Dag Wulsten[3], Hendrik Napierala[1], Johann Pratschke[1], Igor M. Sauer[1,*], Karl H. Hillebrandt[1,6,7], Benjamin Struecker[4,6,7]

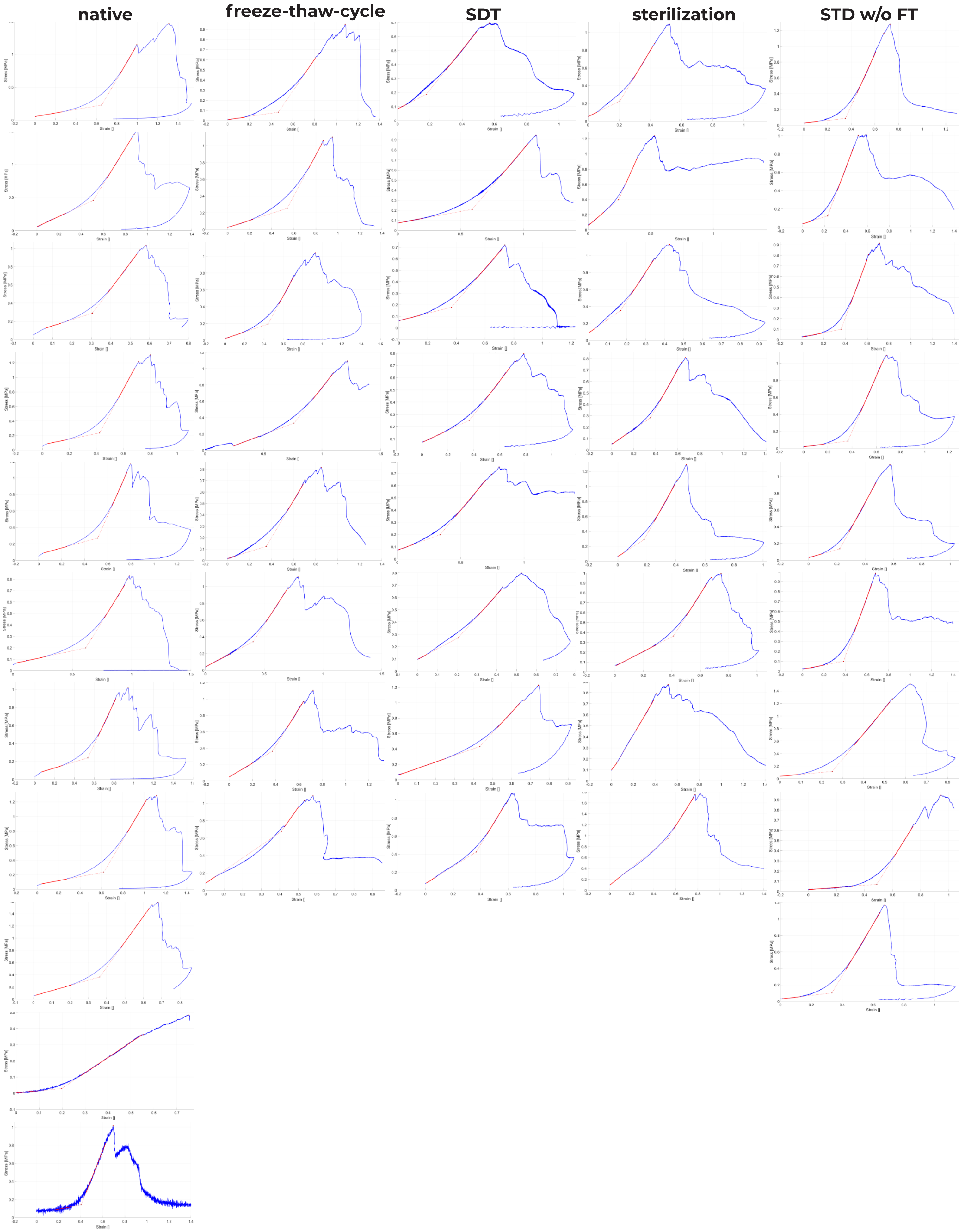

Supplement: Supplementary file 1 — Additional file 1. Shows complete data of the mechanical experiment including stress-strain curves and negative control images for histological staining. [file 13036_2021_266_MOESM1_ESM.pdf]
